# qSR: a quantitative super-resolution analysis tool reveals the cell-cycle dependent organization of RNA Polymerase I in live human cells

**DOI:** 10.1038/s41598-018-25454-0

**Published:** 2018-05-09

**Authors:** J. O. Andrews, W. Conway, W -K. Cho, A. Narayanan, J -H. Spille, N. Jayanth, T. Inoue, S. Mullen, J. Thaler, I. I. Cissé

**Affiliations:** Department of Physics, Massachusetts Institute of Technology, 77 Massachusetts Avenue, Cambridge, MA 02139 USA

## Abstract

We present qSR, an analytical tool for the quantitative analysis of single molecule based super-resolution data. The software is created as an open-source platform integrating multiple algorithms for rigorous spatial and temporal characterizations of protein clusters in super-resolution data of living cells. First, we illustrate qSR using a sample live cell data of RNA Polymerase II (Pol II) as an example of highly dynamic sub-diffractive clusters. Then we utilize qSR to investigate the organization and dynamics of endogenous RNA Polymerase I (Pol I) in live human cells, throughout the cell cycle. Our analysis reveals a previously uncharacterized transient clustering of Pol I. Both stable and transient populations of Pol I clusters co-exist in individual living cells, and their relative fraction vary during cell cycle, in a manner correlating with global gene expression. Thus, qSR serves to facilitate the study of protein organization and dynamics with very high spatial and temporal resolutions directly in live cell.

## Introduction

### qSR: quantitative Super Resolution analysis software

We have developed qSR, a software package for quantitative super-resolution data analysis. qSR integrates complementary algorithms that together form a unique tool for the quantitative analysis of single molecule based super-resolution—PALM^1,2^ and STORM^3^—data from living cells. The input for qSR is a single-molecule localization dataset, and the prior image processing can be performed with popular open-source software like ImageJ^4–6^. qSR readily accepts as inputs the files generated by super-resolution localization plug-ins in ImageJ, including QuickPALM^7^, or ThunderSTORM^8^ which are freely available as add-ons to ImageJ.

Recent open software packages integrate tools for visualization, molecular counting and density based clustering^9–12^. However, these tools do not readily utilize temporal dynamics of protein clustering in living cells^13,14^. Thus a major feature in qSR, which to our knowledge has not been present in any previous analytical package^9–12^, is the integrated toolset to analyze the temporal dynamics underlying live cell super-resolution data.

In qSR, we have added some established complementary algorithms for pair-correlation analysis and spatial clustering^15–18^ which we found most useful while performing temporal dynamic analyses. One example includes a new application of FastJet^19–21^, a cluster analysis package developed by the particle physics community.

**Figure 1:**
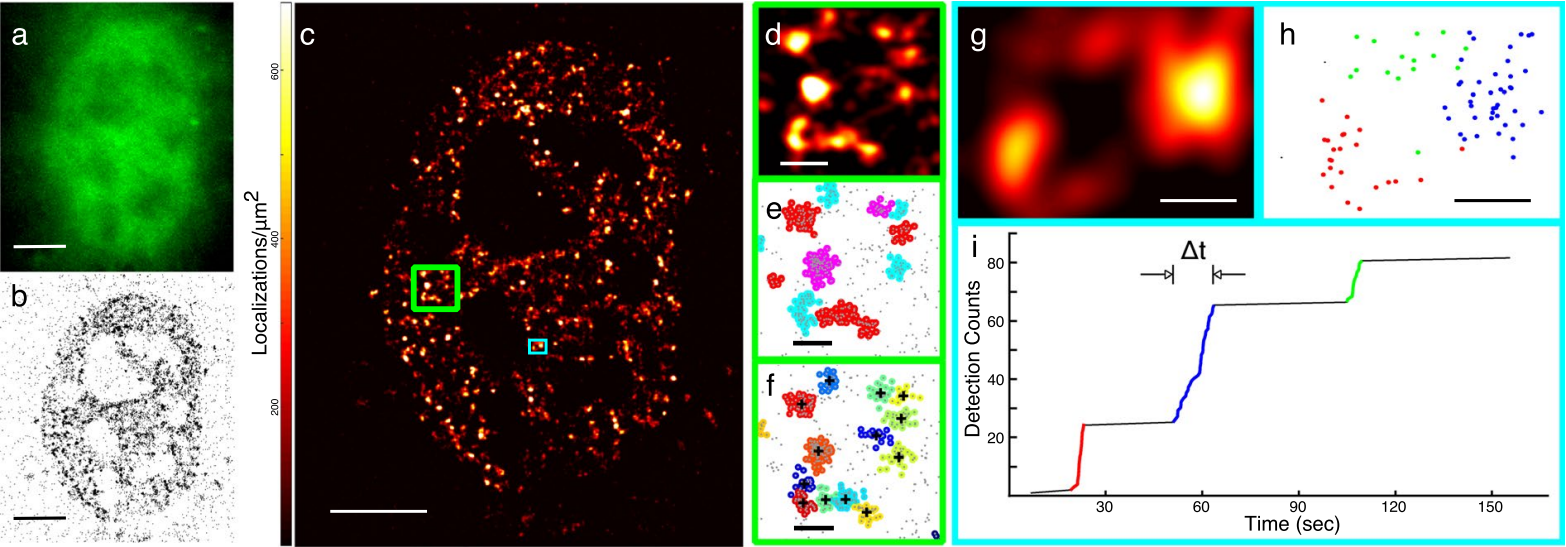
qSR facilitates analysis of the spatial organization and temporal dynamics of proteins in live cell super-resolution data. (**a**–**c**) Conventional fluorescence image, pointillist image, and super-resolution reconstruction image of RNA Polymerase II inside a living cell. (**d,e**) Spatial clustering of the data within the region highlighted in the large green box shown in (**c**) is performed using the DBSCAN algorithm embedded in qSR. (**f**) Spatial clustering of the same region is performed using the FastJet algorithm embedded in qSR. (**g**–**i**) Time-correlation super-resolution analysis (tcPALM) reveals temporal dynamics within a region of interest (ROI) shown in (**g**), and highlighted in the small cyan box in (**c**). In (**i**), for the selected ROI, a plot of the cumulative number of localizations as a function of time is represented. Localizations belonging to the three temporal clusters highlighted in (**i**) are plotted spatially in their corresponding (red, blue, green) colors in (**h**). Clusters of localizations which are grouped by time in **(i)** are also distinctly clustered in space. Scale Bars: (**a**–**c**) 5 µm; (**d**–**f**) 500 nm (**g,h**) 200 nm.

We first test qSR on live cell localization data of endogenously labeled RNA Polymerase II (Pol II) in mouse embryonic fibroblasts, which is known to form transient clusters^22^ [Fig. 1(a)]. We labeled Pol II by fusing Dendra2^23^, a green-to-red photo-convertible fluorescent protein, to the N terminus of RPB1, the largest subunit of Pol II. The pointillist data obtained from single-molecule based super-resolution microscopy techniques—such as photoactivated localization microscopy (PALM)^1,2^, stochastic optical reconstruction microscopy (STORM)^3^ and direct STORM^24^—can be imported into qSR for visualization and analysis [Fig. 1(b)]. Super-resolution images can be reconstructed, and represented in a red-hot color-coded image, by convolving the point pattern of detections with a Gaussian intensity kernel corresponding to the localization uncertainty [Fig. 1(c)].

In addition, qSR enables the quantitative analysis of the spatial distribution of localizations. The qSR analysis tools provide the user with both a summary of detected clusters, including their areas and number of detections, and a global metric of the distribution of sizes via the pair correlation function. For identifying spatial clusters, we have implemented both centroid-linkage hierarchical clustering using FastJet^19–21^ illustrated in Fig. 1(f), and density-based spatial clustering of applications with noise (DBSCAN)^25^ as illustrated in Fig. 1(e).

qSR adopts time-correlated super-resolution analyses—for example tcPALM^13,14,26,27^—to measure the dynamics of sub-diffractive protein clustering in living cells. In live cell super-resolution data, when clusters assemble and disassemble dynamically, the plots of the temporal history of localizations in a cluster show temporal bursts of localizations [Fig. 1(g–i)]. The apparent cluster lifetime and burst size can then be measured, and other clustering parameters, including clustering frequency, can be calculated^13,14^.

For a sample data set, and step by step instruction on how to perform tcPALM please see the user’s guide in the Supplementary Information, section B.1. It is important to ensure that apparent bursts of detections are not due to long-lived single molecules. Therefore, at minimum, control experiments with fixed cells expressing the fluorophore alone (i.e. unfused to any other protein) should be performed to characterize the temporal profile of individual molecules (Supplementary Information section B.3). We note that there are other super-resolution approaches, such as STED^28,29^, and structured illumination microscopy^30,31^ that are not fundamentally based on single-molecule localizations, and the current implementation of qSR is not adapted for those datasets.

### Organization and dynamics of RNA Polymerase I in live human cells

To test the facility of qSR to enable novel quantitative studies with high resolution in living cells we sought to investigate the spatiotemporal organization of RNA Polymerase I (Pol I). Pol I is the molecular complex responsible for the production of ribosomal RNA.

The organization and dynamics of molecular processes inside the nucleolus, the sub-nuclear domain dedicated to ribosome biosynthesis, are emerging as a key intersection point for the regulation of many cellular functions. To balance the need for cellular growth with the energetic cost of ribosome biosynthesis, cells have evolved complex regulatory mechanisms to modulate ribosomal biogenesis in response to the availability of nutrients and growth factors^32,33^. Similarly, stresses that affect cellular homeostasis generally result in signaling cascades that regulate ribosome biogenesis by controlling transcription by Pol I^34–36^. Pol I is thus thought to be a promising target for the treatment of tumors^37,38^ given its central position at the convergence of many different pathways controlling cell proliferation and stress. Yet, despite the central position of Pol I regulation in coordination of cellular processes, it remains unclear how Pol I organization and dynamics may depend on, or reflect cellular demand for ribosomal RNA transcription.

Previous studies in living cells^39^ using fluorescence recovery after photobleaching (FRAP) and kinetic modeling on FRAP data suggested the existence of multiple subpopulations of Pol I interactions with the ribosomal genes. In fact, further studies suggested that ribosomal transcriptional regulation occurs via cell-cycle dependent modulation of these kinetics by altering the availability of a transcription factor (SL1) thereby changing the assembly efficiency of the ribosomal transcription complex^40^. Ensemble-averaging techniques like FRAP, however, are not readily amenable to direct visualization of co-existing dynamic subpopulations and characterization of how each subpopulation putatively changes to help balance cellular progression through different cycles.

Here we use single-molecule based super-resolution to directly measure dynamics of endogenous Pol I in living cells. We fused the catalytic subunit of Pol I, RPA194, to a photoconvertible fluorescent protein, Dendra2^23^, using the CRISPR/Cas-9 genome editing system^22,41–44^ in a human osteosarcoma (U2OS) cell line to create a heterozygous, endogenously labeled cell line. Upon illumination with 405 nm light, the emission maximum of Dendra2 converts from green to red^23^ allowing for imaging of single RPA194 molecules in live cells. We employed quantitative super-resolution imaging on this endogenously labeled Pol I cell line to investigate Pol I organization and dynamics in live cells^13,14^.

With this endogenously labeled Pol I cell line, we used time-correlated photoactivated localization microscopy (tcPALM) to investigate the real time, super-resolution dynamics of Pol I^13,14^. In single-molecule localization based super-resolution techniques such as PALM or STORM a final image is reconstructed based on precise localizations of a sparse, stochastically activated set of fluorophores^1–3^. Time-correlated super-resolution analysis extracts protein dynamics from a super-resolution reconstruction. Since the number of localization events in a temporal window can serve as a measure of local concentration (provided a sufficiently large number of molecules activates within the window), super-resolution acquisition in live cells can provide a measure of the relative spatial and temporal fluctuations of the labeled proteins.

## Results

### Investigation of Pol I cluster dynamics in the interphase nucleus

**Figure 2:**
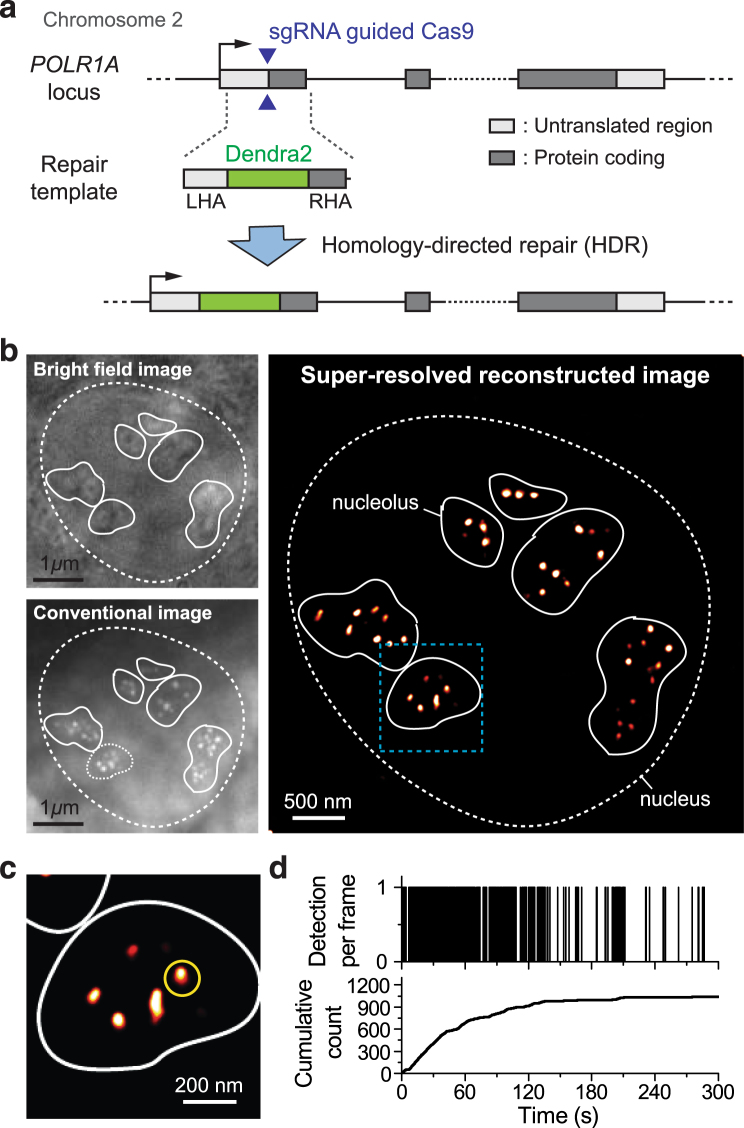
Interphase Organization and Dynamics of RNA Pol I. (**a**) A homologous donor vector containing the Dendra2 fluorescence protein flanked by two 500 bp homologous arms. When co-transfected with a plasmid expressing Cas9 along with a targeted sgRNA, homology directed repair at the protospacer adjacent motif (PAM) cut site induces insertion of the Dendra2 sequence onto the N-terminus of the RPA194, the largest subunit of RNA Pol I. We imaged a total of 77 cells on seven separate days. (**b**) Bright-field and conventional fluorescence imaging of the pre-converted state of Dendra2-RPA194 in a U2OS cell line. The nucleus is demarcated with a dashed line while the contours of the nucleoli are demarcated with a solid white line. The polymerase appears to cluster in distinct foci within the nucleoli. (**c**) Super-resolution reconstruction of the Dendra2-RPA194. At the super-resolution level, foci remain visible. (**d**) A sample time trace of the cluster marked in yellow in the super-resolution image. In the cumulative trace, the detections show an initial linear slope indicating that the cluster was pre-existing and slowly level off suggesting that the cluster is stable.

We began by investigating the real-time dynamics of Pol I during interphase. We imaged a total of 77 individual cells from our Dendra2-RPA194 cell line on seven separate days. Consistent with previous observations^39^ we find that Pol I is localized in distinct foci within the nucleoli. Many of these foci are directly visible by conventional live cell imaging [Fig. 2(b)].

Examining the loci with live cell super-resolution imaging [Fig. 2(c)] and quantifying the dynamics of individual loci using tcPALM reveals that these foci are stable [Fig. 2(d)]. The presence of a cluster is evident in a tcPALM time trace as a steady stream of localizations in the number of detection per frame, or a slope in the cumulative plot. For a stable cluster, the slope is highest from the beginning acquisition (t = 0), indicating that the cluster existed even before image acquisition started. Then, a gradual plateau in the tcPALM time trace suggests that the cluster is still present, but the pool of photoconvertible fluorescent proteins is being gradually depleted by activation and bleaching during the imaging process. The timescale of photoactivation (minutes) determines how quickly the pool of fluorescent proteins in depleted. Consequently, clusters that disassemble more slowly that the activation rate will appear stable in tcPALM.

The presence of multiple, stable sub-nucleolar Pol I clusters during interphase is consistent with a picture of the interphase nucleolus sub-organized into distinct regions including punctate dense transcriptional centers (with high Pol I concentration) separated by a surrounding granular component^45–48^.

### Dynamics of Pol I clustering upon transcription inhibition with CX-5461

Next, we sought to investigate Pol I dynamics at low transcription levels using drug inhibition. We inhibited Pol I initiation with a small molecule inhibitor CX-5461. CX-5461 is a synthetic compound that selectively inhibits Pol I without affecting other transcription (for example mRNA transcription by RNA Polymerase II)^49,50^. In the canonical Pol I recruitment pathway, the factor UBF binds to the rDNA promoter and recruits SL1^32^. SL1 then recruits the downstream transcription factors and associated machinery needed to bind Pol 1 to the promoter. CX-5461 is thought to act selectively on Pol 1 by inhibiting SL1, thereby preventing Pol I initiation. The drug has additionally been observed to induce autophagy and prevent cell growth and division.

**Figure 3:**
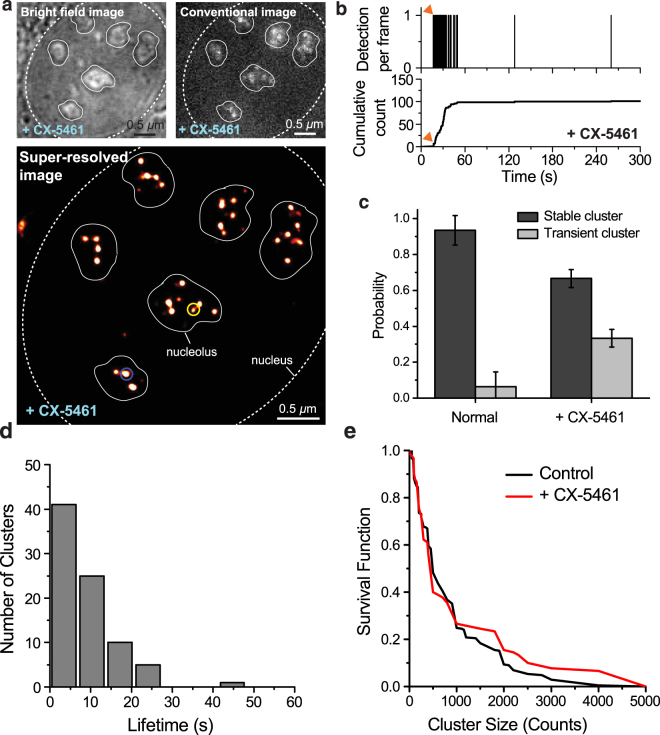
Pol I Response to CX-5461 Initiation Inhibition. (**a**) Bright-field, conventional and super-resolution reconstruction images of the Dendra2-RPA194 U2OS cell line after 48 hours of treatment with CX-5461. Sparse foci appear against a stronger background like in the untreated, interphase nucleus. Many dimmer foci that were not visible in the conventional image appear in the super-resolution reconstruction. (**b**) A sample time trace of a transient cluster (yellow circle in panel a) in the CX-5461 treated nucleus. The burst of detections occurs after a delay (~15 s, red arrow) after the beginning of acquisition, then abruptly stops, signatures of a transiently lived cluster (Supplementary Fig S3). (**c**) The fraction of stable vs. transient clusters in control and CX-5461 treated cells. (**d**) Histogram of transient cluster lifetimes in the CX-5461 treated cells. The mean lifetime is 8.7 ± 0.7 s. (**e**) Survival curve of stable clusters sizes for control and CX-5461 treated clusters. We analyzed a total of 10 cells imaged on three separate days. The cluster size is measured in counts, i.e., the number of localizations within the cluster.

We incubated the cells in 2 μM CX-5461 for 48 hours before performing live cell super-resolution experiments. We imaged a total of 29 cells treated with CX-5461 on three separate days. The initiation-inhibited cells also show clusters in the super-resolution reconstruction (Fig. 3A).

The time traces of individual clusters in drug-treated cells, however, are highly dynamic. In contrast to the stable clusters observed in untreated cells (Fig. 2), many of the initiation-inhibited clusters are transient [Fig. 3(b)] while others are stable (Supplementary Fig. S2). In each cell, we saw a mix of both transient and stable clusters. We can identify a region of interest where a cluster existed at some point during acquisition by examining the final PALM image. In tcPALM localization time traces of these regions, transient clusters are evident by time traces characterized with virtually no localizations at the beginning, suggesting that the cluster had not yet formed; then localization frequency suddenly increases, indicating a sudden increase in local concentration of photoconvertible proteins, and suggesting the cluster assembled in real time (for more information on the transient/stable distinction, see Supplementary Fig. S3). The high frequency of localization then ceases abruptly suggesting that the cluster has rapidly disassembled [Fig. 3(b)]. The number of detections in this transient jump cannot be accounted for by single molecule photophysics, so multiple polymerases multiple proteins were likely recruited to the locus^13,51,52^. Together, the features in the example time trace in Fig. 3(b) suggest that Pol I clusters become transient, assembling and disassembling dynamically, upon transcription inhibition. The transient Pol I clusters we observe have a mean lifetime of 8.7 ± 0.7 s [Fig. 3(d)].

To ensure that the transient clusters we observed were in fact distinct from the stable Pol I foci, and not simply small, but stable, Pol I clusters, we performed simulations of Dendra2 photo-physics. We compared the progression of polymerases in a stable cluster to a cluster of polymerases that only associated for a brief time. The details of our simulation scheme can be found in *Materials and Methods*. A comparison of the results of our simulation for both the stable clusters we observed in interphase and the transient clusters we observe when we inhibit initiation with CX-5461 can be found in Supplementary Fig. S2. Our simulations suggest that transient features observed in the tcPALM traces are not compatible with stable foci given the photophysics of Dendra2.

We quantified the fraction of transient clusters in CX-5461 treated and control cells [Fig. 3(c)]. We observe that the drug treated cells contain substantially more transient clusters than the control cells. Remaining stable clusters may result from the drug failing to turning off all ribosomal transcription; consistent with this interpretation we find that the remaining stable clusters are similar in size to those found in the control cells [Fig. 3(e)] with no significant difference in the distributions.

### Cell-cycle dependence of Pol I clustering

We investigated the dynamics of Pol I clusters in various stages of the cell cycle. Since rDNA transcription ceases when chromosomes are condensed, we were curious if we would observe a similar increase in transient clustering during mitosis. We hoped to then observe how clustering dynamics changes as cells grew and replicated their DNA in preparation for division. We blocked cells in S phase by halting the cell cycle using a double thymidine block^53^. Releasing these cells from thymidine produces synchronized cells in M and G1 phases. We imaged 29 M phase cells, 27 G1 phase cells and 30 S phase cells on nine separate days.

**Figure 4:**
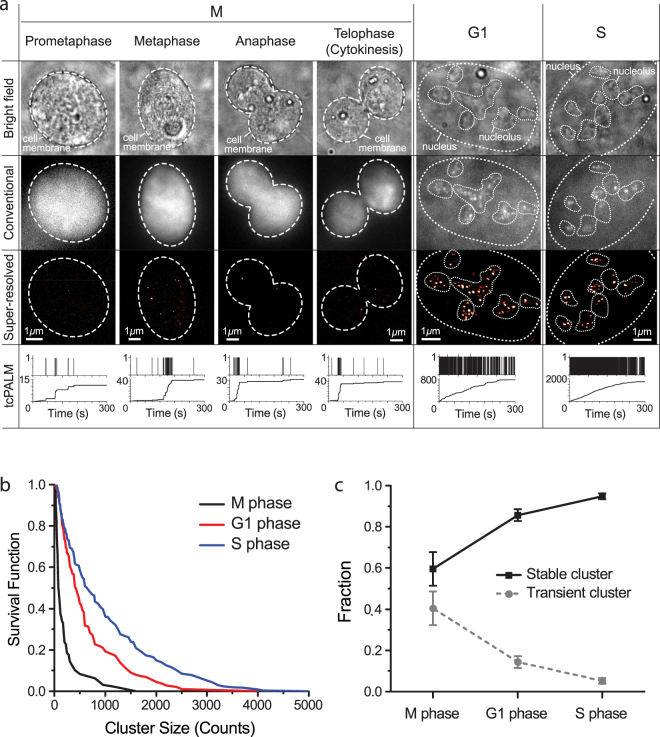
Cell-cycle dependent organization and dynamics of RNA Polymerase I. (**a**) Bright-field, conventional, super-resolution images and tcPALM time traces of M, G1 and S phase cells. The upper panel of the tcPALM trace represents detections per frame; the lower panel represents the cumulative detection count. Magnified tcPALM time traces are available in Supplementary Fig. S4. (**b**) Survival curve of stable cluster size in M, G1 and S phase cells. The cluster size is measured in counts, i.e., the number of localizations within the cluster. (**c**) Portion of stable and transient clusters in M, G1 and S phase cells. We imaged a total of 29 M phase cells, 30 S phase cells and 27 G1 phase cells on nine separate days.

The Pol I spatial organization in M phase differs substantially from interphase Pol I organization. Unlike in interphase where we observed foci of multiple, adjacent Pol I clusters in nucleoli, we see no similar groupings of Pol I clusters in M phase. In fact, we observe fewer and spatially distinct Pol I clusters in M phase [Fig. 4(a)]. tcPALM analysis of cells imaged in M Phase shows a mixture of both stable and transient Pol I clusters [Fig. 4(b)].

The observation of a mixture of stable and transient Pol I clusters agrees well with previous descriptions of mitotic rDNA organization^32,45,54^. During mitosis, the chromosomes condense and transcription of rDNA ceases^55^. Previously bound Pol I molecules are thought to remain bound to the rDNA genes during this transcriptionally silent period^45^. Novel Pol I binding events, however, are believed to be inhibited, putatively through Runx2/SL1 dependent transcription inhibition^54^. The stable clusters in M phase are likely attributable to previously initiated and elongating polymerases that remain bound to the rDNA genes during mitosis. The transient clusters, however, may be due to the absence of the SL1 as we observed in our drug inhibition experiments in Fig. 3.

To quantify the differences in Pol I cluster dynamics between different phases of the cell cycle, we evaluated both the portion of transient and of stable clusters and the size distribution of the stable clusters [Fig. 4(b)]. We found that M phase displays the highest fraction of transient clusters, and the fraction of transient clusters decreases as cells progress into G1 and later S phase. Similarly, we quantified the relative intensity of stable clusters.

We observe more detections per stable clusters as cells progress out of M phase into G1 and S (Fig. 4(c) and Supplementary Fig. S4). Translating number of detections into number of molecules is an intricate task in super-resolution data due to non-trivial single-molecule photophysics. Nonetheless, more detections likely indicate that more polymerase molecules are present in the clusters. The cluster size distribution as measured by the number of localization from the stable clusters between M, G1 and S phase was noticeably different [compare different color graphs in Fig. 4(b)]. Thus, Pol I clusters tend to become more intense (i.e., larger by the number of molecules per foci) and more stable as the cells grow and synthesize new DNA in preparation for division.

We note that the observation of larger, more stable clusters corroborates previous interpretations from FRAP data showing longer retention times for individual polymerase subunits and the SL1 transcription factor in S phase than G1^40^. The increase in Pol I cluster stability and size also agrees with previous observations that Pol I activity increases as cells progress through G1 and peaks in S phase^35^.

The simultaneous presence of a distinct transient population of RNA Polymerase I, however, could not have been directly inferred from previous measurements. Moreover, our observation of the onset of transient clustering from both drug-induced and cell-cycle driven inhibition of SL1 suggests that the fraction of stable versus transient clusters may be interpretable as the relative fraction of actively transcribed rDNA genes versus inactive, silent genes. This measure thus likely reflects the overall activity of the rDNA genes.

## Conclusion

Taken together, our live cell super-resolution data paint a dynamic picture of cell cycle dependent Pol I clustering: Pol I forms stable foci in the nucleolus where rDNA genes are clustered and when many polymerases are actively transcribing the rDNA genes. When initiation is inhibited, Pol I may still cluster, albeit very transiently. Transcriptionally active nucleolar centers thus appear as stable clusters of polymerase while inactive centers appear as transient clusters.

The type of quantitative live cell super-resolution analysis can be done in principle for any protein that can be fused to a GFP-like fluorescent tag. The real time quantitative characterizations of Pol I, and the direct observation of different subpopulations (transient versus stable) of Pol I clusters in living cells, serves to illustrate the types of novel insights that can be drawn from their own studies using the qSR analytical software.

qSR is provided as an open source software package with a graphical user interface that a researcher can download and use directly. For illustration, and to help new users quickly familiarize themselves with qSR, we provide the live cell super-resolution data represented in Fig. 1 as sample data^22^ packaged along with the software, as well as step by step instructions in Doc. S1. Installation instructions and protocols for performing the analyses are also provided in Doc. S1. qSR is maintained and distributed for free on www.github.com/cisselab/qSR. By providing qSR as an open source platform, we hope to encourage other users in the community to develop and integrate any multitude of algorithms, features and modules for qSR, as the field evolves. For examples approaches for counting molecules in clusters^51,56,57^, could be complimentary extensions beyond the current scope of qSR. Users are prompted to directly cite the original publications for any third-party algorithm they use within the qSR platform.

## Materials and Methods

### CRISPR-Cas9 mediated insertion of Dendra2 onto the N-Terminus of RPA194

A human osteosarcoma (U2OS) cell line with an endogenous N-terminal Dendra2 insertion in the RPA194 gene was generated via the CRISPR/Cas9 system^41–44^.

The cells were transfected with both pSpCas9(BB)-2A-Puro (px459) V2.0, containing an sgRNA targeted to start of the RPA194 gene (5′-TTCAGCCGAATACATCCCCGAAGG-3′), and a homology directed repair template. sgRNA sequences were generated via the online CRISPR toolbox (crispr.mit.edu) and then cloned into the px459 vector with one step digestion ligation (Supplementary Table S1)^54,58^. All experiments and validation were performed on the cell line transfected with sgRNA1. Of the remaining guides, only sgRNA5 showed successful insertion. pSpCas9(BB)-2A-Puro (px459) V2.0 was a gift from Feng Zhang (Addgene plasmid # 62988).

The homologous donor sequence was synthesized by Life Technologies to contain a 500 bp left homologous arm, the 690 bp Dendra2 insertion and a 500 bp right homologous arm. Silent mutations were introduced into the protospacer adjacent motif (PAM) sites of 6 potential sgRNAs in the right homologous arm to ensure the repair template was not degraded by the Cas9 system. The repair template was PCR amplified to prepare a linear fragment for transfection (Supplementary Table S2).

The linear homologous repair template and the px459 plasmid containing the sgRNA insert were transfected into the U2OS cell line using the x-tremeGENE 9 transfection reagent from Sigma-Aldrich. Cells were left to incubate at 37 °C for 24 hours. The cells were then allowed to recover, and sorted via FACS at the Koch Institute Sorting facility to isolate cells expressing the Dendra2 insertion for imaging.

### Cell Culture Protocols

The Dendra2-Pol I cells and Dendra2-Pol II cells^22^ were cultured in Dulbecco’s Modified Eagle Medium with Glutamax (DMEM with Glutamax) from Thermo Fisher (10567). The media contained 10% fetal bovine serum from Gibco (26140-079, US Origin, Qualified) and an antibiotic mixture at a final working concentration of 10 U/mL penicillin and 10 μg/ml streptomycin from Gibco (15140). Cells were incubated at 37 °C with 5% supplemental CO2.

### Initiation Inhibition with CX-5461

Cells were incubated with 2 μM CX-5461 (Selleckchem 1138549-36-6). CX-5461 is a potent, selective inhibitor of SL1, a transcription factor associated with Pol I binding to the rDNA gene^49,50^. CX-5461 was stored at 10 mM in a stock solution of 50 mM NaH2PO4 (pH 4.5) and added directly to imaging dishes for treatment.

### Cell Cycle Synchronization via a Double Thymidine Block

Cells were synchronized using a double thymidine block approach previously reported^53^. Cells were treated with 2 mM of thymidine from Sigma Aldrich (T1895-1G) dissolved in the 10% FBS DMEM media previously described and incubated at 37 °C for 15 hours to arrest cells in S phase. Following this initial S phase arrest, cells were released via removal of the thymidine medium and allowed to progress for 9 hours to ensure all cells had passed out of S phase. Cells were then re-incubated with 2 mM thymidine to synchronize cells at the G1/S junction. To produce cells in S phase, cells were imaged ~one hour after release from thymidine control. For M phase, cells were imaged between 9 and 12 hours after release from thymidine and subject to visual confirmation of ongoing mitosis (i.e., rounded cell shape, finger-like projections, cell division, etc.). For G1 cells, cells were imaged 15 hours after thymidine release.

### Super-Resolution Imaging

Cells were imaged on a homebuilt super-resolution setup, comprised of a Nikon Eclipse TI microscope equipped with a 100x oil immersion objective (NA 1.40) (Nikon, Tokyo, Japan) and lasers and filter sets for 405 nm, 488 nm, and 561 nm illumination. During imaging, cells were kept at 37 °C in an incubator set atop the objective (InVivo Scientific, St. Louis, MO). Images were captured on an Andor iXon Ultra 897 EMCCD camera at a rate of 60 ms/frame at an EM-gain of 900. Camera image acquisition and control was performed using Micro Manager 1.4^59^. Cells were held steady in the z-direction using the Perfect Focus System of the Nikon Microscope during image acquisition. Conventional fluorescence imaging was achieved via 50 ms of exposure with a 488 nm laser.

For PALM imaging, excitation (561 nm) and activation (405 nm) lasers were combined, expanded then focused on the sample. These beams were expanded using an achromatic beam expander (AC254-040-A and AC508-300-A, from THORLABS, Newton, NJ) and refocusing was performed with an achromatic converging lens (#45–354, from Edmund Optics, Barrington, NJ). Power levels were controlled both by directly varying the initial laser intensity and through an AOTF, and measured directly at the top of the objective lens closest to the sample slide.

### Image Analysis and tcPALM

We analyzed images following the general scheme previously described in depth in Cho *et al*. and Cissé *et al*.^13,14^. Individual Pol I molecules were localized using a modified MTT localization algorithm^60^. Super-resolution images were generated from a Gaussian spreading of the localizations determined by the MTT program. We then analyzed these localizations using qSR. Step-by-step instructions of how to use qSR can be found in Supplementary Information.

### tcPALM Simulation Using Hidden Markov Models

We simulated tcPALM acquisitions using the hidden Markov model package in MATLAB^61^. As in previous work, we considered a four-state model (pre-converted, fluorescent, dark and bleached) for Dendra2^22,51^. Initially, all of the Dendra2 fluorophores are in a dark, pre-converted states. As acquisition proceeds, the Dendra2 are stochastically photoactivated by the 405 nm laser at a rate k_on_. This photoactivation converts Dendra2 into a fluorescent state. Since Dendra2 is a blinking dye, this fluorescent state exists in dynamic equilibrium with a dark state distinct from the bleached state. The fluorescent state can convert to the dark state with a rate k_dark_ and recover back to the fluorescent state with rate k_rec_. In contrast, photobleaching is irreversible. The fluorescent state is then photobleached by the 561 nm laser at a rate k_bleach_ and proceeds, into the bleached state. Of these four states, only the post-conversion fluorescent state is directly observable during tcPALM acquisition.

Previous work in our lab and others^13,51,52^ has assessed the photophysical parameters of Dendra2 in U2OS. Since we engineered our cells from the same stock and used the same microscope setup with the same 561 nm laser power, we felt confident using the previously determined values for k_dark_, k_rec_ and k_bleach_ (k_dark_ = 9.61/s, k_rec_ = 2.33/s and k_bleach_ = 3.0/s). We did, however, use a higher photoactivation power than previous experiments, so we measured k_on_ independently. We analyzed the tcPALM trace of the entire nucleus and fit our trace to an exponential model with a constant, linear noise term: N = N_0_ (1 − exp(−k_on_ * t)) + B * t. From this analysis, we found that k_on_ = (1/43 s).

We assigned each of these four states to a state of a hidden Markov model. Given the progression scheme described above, we used the transition matrix:

$${\rm{T}}=\begin{array}{cccc}[(1-{{\rm{k}}}_{{\rm{o}}{\rm{n}}}\,\ast \,{\rm{\Delta }}t) & {{\rm{k}}}_{{\rm{o}}{\rm{n}}}\,\ast \,{\rm{\Delta }}t & 0 & 0\\ 0 & (1-{{\rm{k}}}_{{\rm{d}}{\rm{a}}{\rm{r}}{\rm{k}}}\,\ast \,{\rm{\Delta }}t-{{\rm{k}}}_{{\rm{b}}{\rm{l}}{\rm{e}}{\rm{a}}{\rm{c}}{\rm{h}}}\,\ast \,{\rm{\Delta }}t) & {{\rm{k}}}_{{\rm{d}}{\rm{a}}{\rm{r}}{\rm{k}}}\,\ast \,{\rm{\Delta }}t & {{\rm{k}}}_{{\rm{b}}{\rm{l}}{\rm{e}}{\rm{a}}{\rm{c}}{\rm{h}}}\,\ast \,{\rm{\Delta }}t\\ 0 & {{\rm{k}}}_{{\rm{r}}{\rm{e}}{\rm{c}}}\,\ast \,{\rm{\Delta }}t & (1-{{\rm{k}}}_{{\rm{r}}{\rm{e}}{\rm{c}}}\,\ast \,{\rm{\Delta }}t) & 0\\ 0 & 0 & \,0 & 1]\end{array}\,\,\,\,\,\,\,\,\,\,\,\,\,\,\,\,\,\,\,\,\,\,\,\,\,\,$$

We simulated progression of an adjustable number of Dendra2 molecules through the tcPALM acquisition process individually and aggregated the results to simulate cluster dynamics. We used a timestep ∆t of 6 ms and then binned our results into distinct 60 ms bins to simulate our exposure time. If any of the molecules we had simulated were in the fluorescent state at any point in our 60 ms bin, we assumed that we would observe a localization. Within a cluster, if multiple molecules are visible at the same time they will appear as a single spot. Consequently, they will contribute a single localization count. Therefore, in the simulation this is reflected as a single detection count. For stable clusters, we analyzed our simulation result for the entire acquisition window while for transient clusters we only considered our acquisition results within a limited temporal window and set the localizations equal to zero elsewhere.

## Electronic supplementary material

Supplementary Information
