# Supplementary material for: qSR: a quantitative super-resolution analysis tool reveals the cell-cycle dependent organization of RNA Polymerase I in live human cells
Source: Sci Rep. 2018 May 9;8:7424. doi: 10.1038/s41598-018-25454-0 (PMC5943247; doi:10.1038/s41598-018-25454-0)
Supplement: Supplementary file 1 — Supplementary Information [file 41598_2018_25454_MOESM1_ESM.pdf]

Supplementary Information

qSR: a quantitative super-resolution analysis tool reveals the cell-cycle dependent organization of RNA Polymerase I in live human cells

JO Andrews[1], W Conway[1], W-K Cho, A Narayanan, J-H Spille, N Jayanth, T Inoue, S Mullen, JD Thaler and II Cissé*

[1] These authors contributed equally to this work.

Supplementary Table S1: sgRNA sequences targeted *POLR1A* gene

		Sequence (5′ ⇒3′)
sgRNA #1	(forward)	CACCGTTCAGCCGAATACATCCCGA
	(reverse)	AAACTCGGGATGTATTCGGCTGAAC
sgRNA #2	(forward)	CACCGCCGCCGCCAGGGCATGTTCT
	(reverse)	AAACAGAACATGCCCTGGCGGCGGC
sgRNA #3	(forward)	CACCGTCGGCTGAAGAGCTCAAGTA
	(reverse)	AAACTACTTGAGCTCTTCAGCCGAC
sgRNA #4	(forward)	CACCGGTCGGGTAGCGTGCCCAGCC
	(reverse)	AAACGGCTGGGCACGCTACCCGACC
sgRNA #5	(forward)	CACCGCATTTCCTTCGGGATGTATT
	(reverse)	AAACAATACATCCCGAAGGAAATGC

* Red : BbsI restricted DNA overhang sequences

Supplementary Table S2: Repair template for Dendra2 gene knock-in

Homology left arm (5′ UTR of *POLR1A*)	TTGTACCGTCGACAGCCAGCGAACAGCAGATACAACCGCCATCTTTGATTTCTCTGCAGAGCATG<br>TTGGGAAAGCGTGCCTCGACCTTCAGGAAGGTGGGGCGCAGTGGGCGGGGAGCAGGGAACAGCTC<br>CGCTATTGGCTGAAGCGGAAGAAGACGAAAGCAATCATAAAATGGGAGGTTGCAAGCTCATGGTT<br>TGAAAGACTTCGTCACGGAAGCTAAAAGCTCTATACACCCGATTTGCCTCGGAGGAATTTTCCTA<br>AATGATTATTTTGATGTCTTATATATCTTTGATTGTTTTCAAAACAAAAGAGCGAGCAGAGAGTC<br>GTACAACTATTTGTTCCCCCCCCATGTAGAAGTGATCTCATCCACGTAAATGTCGTTCCTGCGAC<br>CGCTTCCGCGCGCAAGCGCACGTTGAATCGCGTGGTGACTCCGGGCTTGAGGTTGAATTAAGAAT<br>AGTCAGGTGGTGAGTGGAACGTCTCTTGGGGTGTCGGAATTCAAAACGGACCTGGAGGATGAACA<br>CCCCGGGAATTAACCTGATCAAGGAGGACATGCGCGTGAAGGTGCACATGGAGGGCAACGTGAAC
Dendra2 sequence	GGCCACGCCTTCGTGATCGAGGGCGAGGGCAAGGGCAAGCCCTACGAGGGCACCCAGACCGCCAA<br>CCTGACCGTGAAGGAGGGCGCCCCCCTGCCCTTCAGCTACGACATCCTGACCACCGCCGTGCACT<br>ACGGCAACCGGGTGTTCACCAAGTACCCCGAGGACATCCCCGACTACTTCAAGCAGAGCTTCCCC<br>GAGGGCTACAGCTGGGAGCGCACCATGACCTTCGAGGACAAGGGCATCTGCACCATCCGCAGCGA<br>CATCAGCCTGGAGGGCGACTGCTTCTTCCAGAACGTGCGCTTCAAGGGCACCAACTTCCCCCCCA<br>ACGGCCCCGTGATGCAGAAGAAGACCCTGAAGTGGGAGCCCAGCACCGAGAAGCTGCACGTGCGC<br>GACGGCCTGCTGGTGGGCAACATCAACATGGCCCTGCTGCTGGAGGGCGGCGGCCACTACCTGTG<br>CGACTTCAAGACCACCTACAAGGCCAAGAAGGTGGTGCAGCTGCCCGACGCCCACTTCGTGGACC<br>ACCGCATCGAGATCCTGGGCAACGACAGCGACTACAACAAGGTGAAGCTGTACGAGCACGCCGTG<br>GCCCGCTACAGCCCCCTGCCCAGCCAGGTGTGGATGTTGATCTC**A**AAGAACATGCCCTGGCGGCG<br>**A**CTGCAGGGCATTTC**A**TTCGGGATGTATTC**A**GCTGA**G**GAGCTCAAGTAAGGAGTTGGTCGGGTAG<br>CGTG**T**CCAGCCTGGATCTTGAGGTCGGCGCCCTAAACTACGATTCCCAGAAGCGCTTTTGCCCAG
Homology right arm (*POLR1A* gene sequence)	TCTATCCTCTTGAGTCCAGGCTTGTCTGGCGCTTGTCCTGCTGGGAGCCGTAGTTCTGGGAACGT<br>TCCTGGGCCGGCGTGGACTGCCTGGATAAGAGGCCGGTGGTGCTGGGGGAAAGAGTTTGCGCTGA<br>GGTAGGGGAATATAAGGGTGGGGAGGGTCCTGGTCTGCAGCGTTATCATCTGCCAGTCTCCTCGT<br>TAACTAACAAGCATTTGTGGAATTAACCATAGTGTACCTGACATGGTTAATTAGGCACGTAGTAA<br>GTTTCCTCTTGTTTGTAAAATGATTGTCCTTCAAGTGTCAGTTCAGGCGTTAACTTCTCTTTGCT<br>AGGACCCTGCTATAAGCTTTATTGAT

* Green : start site of Dendra2 * Yellow : start site of *POLR1A* * **Red bolded letters** : silent mutation

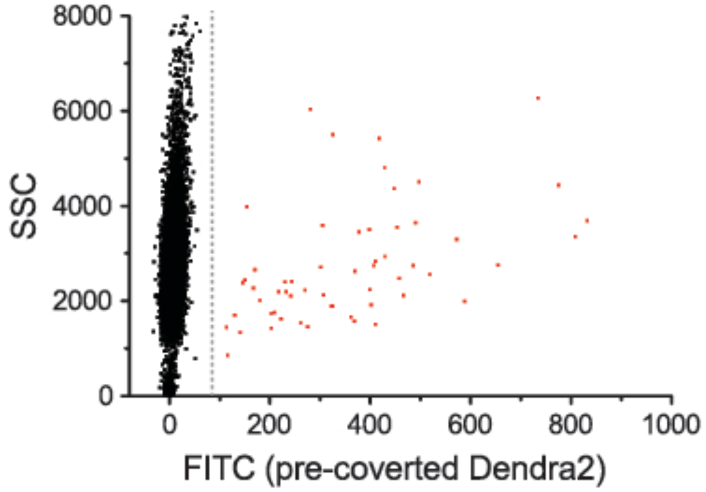

Supplementary Figure S1: Fluorescence activated cell sorting (FACS) dot plots

Cells transfected with sgRNA#1-Cas9 construct along with Dendra2 repair template were sorted using FACS to identify fluorescent cells. A dot plot shows measurements of side scatter (SSC) and fluorescence detection of pre-converted Dendra2 through FITC filter (488-nm excitation) for 10,794 cells. We set a sorting threshold on the maximum intensity detected in wild type cells to isolate successfully transfected cells. 0.67% of the fluorescent cells displaying intensities above the threshold were collected for live cell imaging.

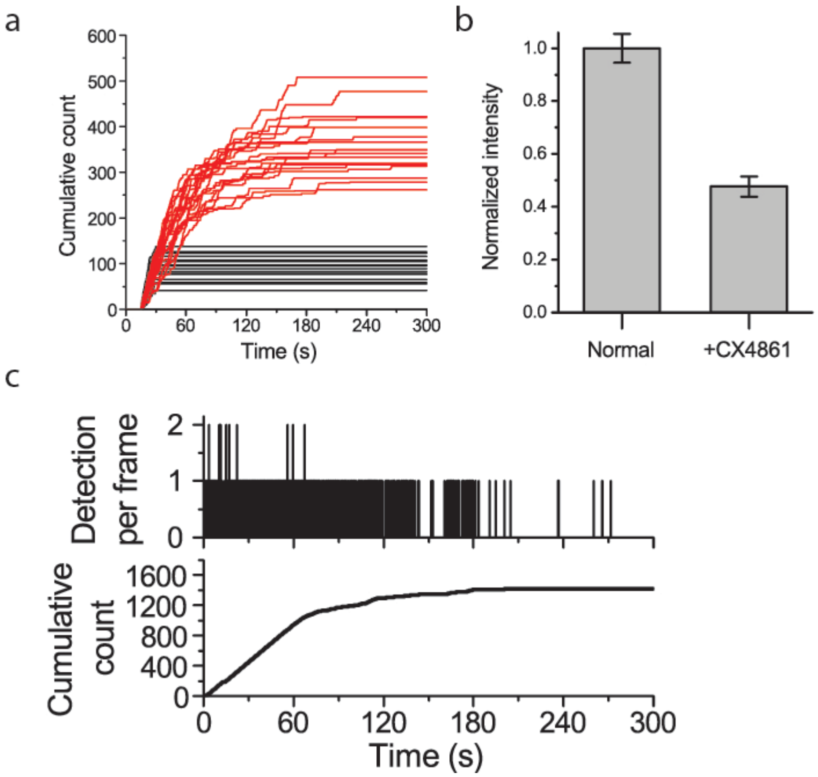

Supplementary Figure S2: Additional quantification of the CX-5461 treated cells

(**a**) tcPALM dynamics for stable and transient clusters were simulated using the hidden Markov model package in MATLAB. We assumed the Dendra2 begins in a dark, pre-converted state and is subsequently activated to a fluorescent state by the 405nm laser. This fluorescent state interconverts in dynamic equilibrium with a transient dark state before it is bleach to a permanent dark state by the 561nm laser. We show simulated tcPALM traces from clusters of 50 molecules that are stable (red) and transient (black). Both sets of clusters begin simultaneously at 15 seconds after acquisition. In contrast to the transient clusters, the stable clusters continue to grow long after the brief transient window has closed and consequently display a higher final total detection count. (**b**) Raw intensities of pre-converted Dendra2-Pol I in normally grown cells and CX-5461-treated cells were measured. N=55 foci from 16 normally grown cells and N=45 foci from 13 CX-5461-treated cells were collected. 200 frames of images were averaged for each cell. Pol I foci of the CX-5461 were less than half as bright as the foci of normally grown cells (I=0.48 ± 0.04; mean ± s.e. of mean). (**c**) A sample time trace of a stable cluster in the CX-5461 treated nucleus [blue circle Figure 3 (a)].

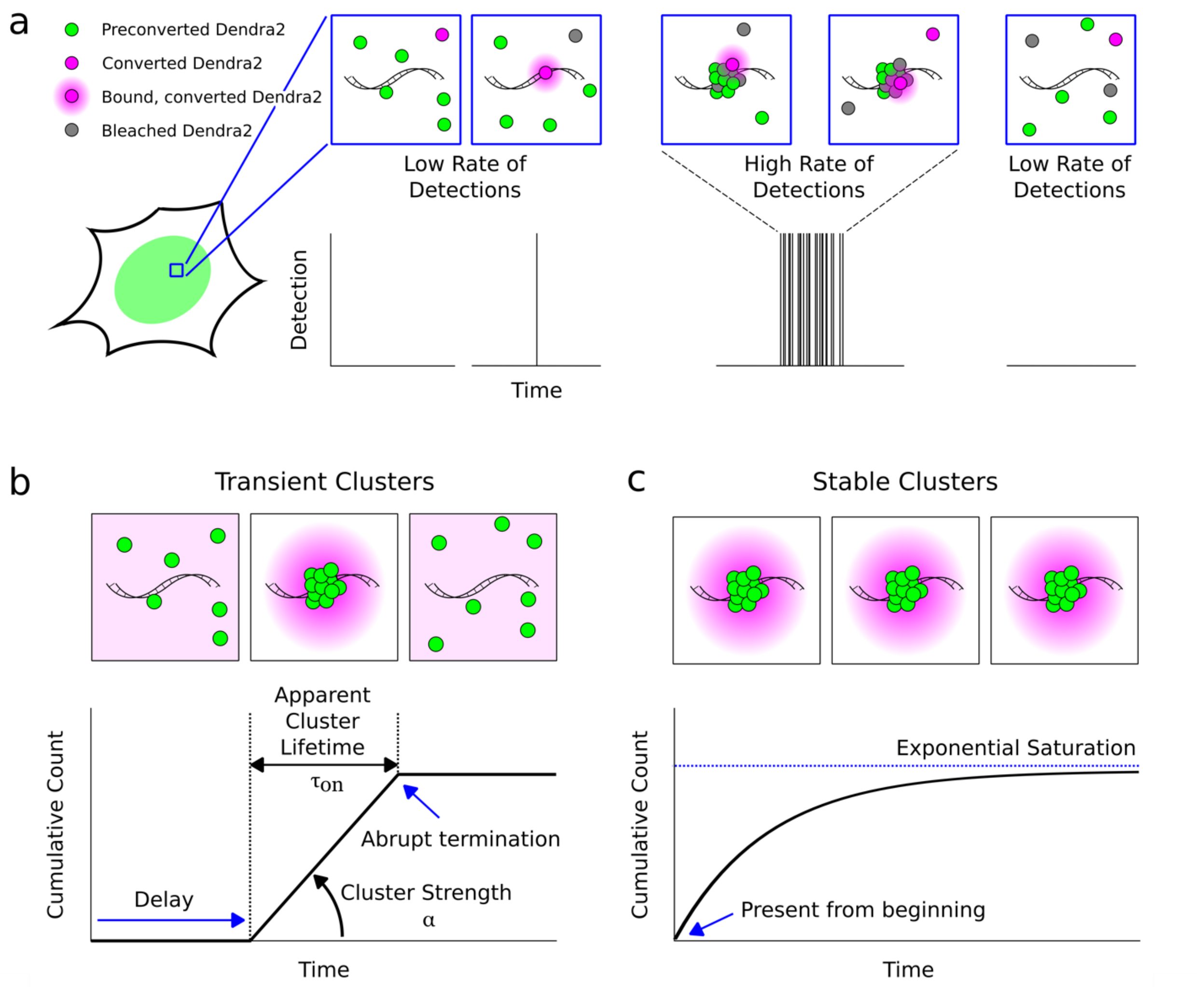

Supplementary Figure S3: Interpreting tcPALM traces.

(a) Illustration of the different fluorescent states of Dendra2- fluorescent molecule and the expected frequency of detections based on local density. Only bound converted molecules contribute to the detections; freely diffusing molecules will not typically be resolved due to motion blur. If multiple bound molecules are present then successive activations result in a high frequency of detections. (b) Illustration of a transient cluster. Transient clusters are characterized by a series of localizations that are correlated in time. In practice, transient clusters are identified (using the graph of the cumulative count of detections) as an increased rate of detections that begins with a delay after the beginning of imaging, and that abruptly terminates. (c) illustration of a stable cluster. Stable clusters exhibit a high rate of detections from the beginning of acquisition, and reach an exponential saturation as the population of pre-converted

molecules is depleted. Clusters that do not exhibit a delay but do display an abrupt termination (putatively transient clusters that assemble around the start of acquisition), or clusters starting with a delay after acquisition then gradually reaching an exponential saturation (putatively clusters that assembled dynamically after start of acquisition then remained stable) were very rare in our experiments.

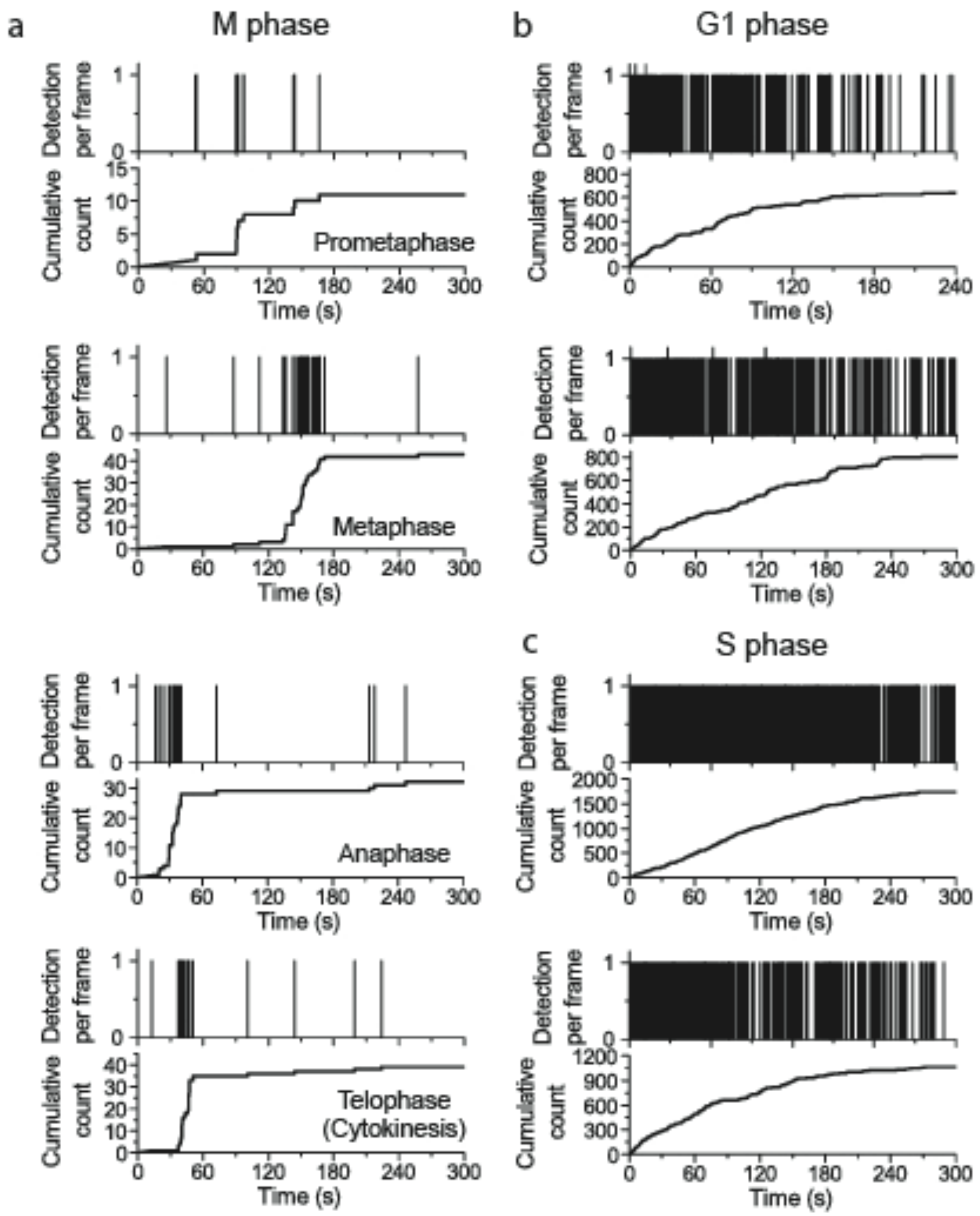

Supplementary Figure S4: Example traces for Dendra2-Pol I in M-, G1- and S-phase

(**a**) Dendra2-Pol I traces in various stages of M-phase reveal transient clusters. (**b**) G1-phase traces show stable clusters. (**c**) S-phase traces show stable clusters. S-phase clusters display more polymerase detections than G1 clusters. This higher detection count corresponds to higher rates of rRNA transcription.

qSR User's Guide

Table of Contents

Section A: Installation Instructions Page 8
A.1: Installing as a stand alone program
A.2: Installing from source code

Section B: Protocols Page 10
B.1: Main Figure 1 Analysis Protocols:
Supplemental Figure 1: The qSR interface.
Supplemental Figure 2: Calling qSR from MATLAB.
Supplemental Figure 3: DBSCAN interface.
Supplemental Figure 4: Schematic Representation of the temporal cluster selection Interface.
B.2: Additional Analysis Protocols
B.2.a: Pair Correlation Analysis
Supplemental Figure 5: Pair correlation analysis.
B.2.b: FastJet Hierarchical Clustering
Supplemental Figure 6: FastJet hierarchical clustering.
B.3: Single Molecule Control Data
Supplemental Figure 7: Characterizing single molecules.

Section C: Input Data Format Page 18
Section D: Software File Structure and Data Representation Page 18
Section E: Summary of Included Algorithms Page 19
Section F: User Feedback and Bug Reports Page 19
References Page 20

Section A: Installation Instructions

A.1: Installing as a stand alone program (recommended)
For most users (including those without a MATLAB license), the simplest installation is through the "stand alone" program that can be downloaded from our github code-sharing portal on the web at www.github.com/cisselab/qSR. Users of the software are invited to ask questions, suggest features, and report bugs by logging issues on www.github.com/cisselab/qSR.

1. Navigate to https://github.com/cisselab/qSR/releases. This paper was drafted with release version v1.1.0.

2. We provide installation tools, with a dialog box providing step by step guidance, for both Windows and Mac OSX operating systems. Download and run the appropriate qSR installer. We also provide sample data, ExampleData_SRL.csv.

   Note: We advise copying the sample data from the downloads folder into a new folder. Otherwise, some users may experience issues accessing data in their downloads folder, particularly when running FastJet clustering.

For most users, the stand-alone program is sufficient. These users can proceed directly to Section B. For users who prefer to install from source, or to run and edit qSR within MATLAB, please proceed to section A.2.

A.2: Installing from source code (alternative)
Alternatively, the source code is made available for users that own a valid MATLAB license. qSR is distributed as an open source platform for the community. Users with expertise are encouraged to contribute, edit, or modify qSR as they see needed. We request that such upgrades be made available to the community by updating the qSR code directly on Github. We will monitor contributed updates and incorporate broadly appealing new features in future releases/upgrades.

The software makes use of the "Statistics and Machine Learning" Toolbox as well as the "Image Processing" Toolbox, so these will need to be installed to run the code. The software is available at www.github.com/cisselab/qSR. Most features of the software can be run after step 1 below. Continue to step 2 if you wish to run pair correlation analysis, and to step 3 if you wish to use FastJet hierarchical clustering for spatial clustering and automated ROI suggestion.

1. Navigate to https://github.com/cisselab/qSR/releases. This paper was drafted with release version v1.1.0. Download the source code zip file and unzip the program. Add the qSR folder, along with all subfolders, to your MATLAB directory.

2. For the pair correlation analysis, our software makes use of code developed by Veatch *et al*.[1] The function get_autocorr is made available in the cited publication as file S1. The code should be downloaded, renamed as get_autocorr.m and added to the qSR software folder.

3. To perform FastJet Hierarchical Clustering, the FastJet[2-4] code must first be compiled. qSR uses the fjcore distribution of FastJet version 3.2.0, a software package developed by the particle physics community for jet finding and analysis at colliders. FastJet is distributed as c++ source code.

a. For Unix operating systems (OSX, Linux), from the command line, first cd into the qSR/SpatialClustering/BioJet/FJCore/fjcore-3.2.0 folder, and then compile using g++ by calling

   g++ -O BioJetsTree.cc fjcore.cc -o BioJetsTreeUnix

   In OSX, the g++ compiler is available as a part of the Apple's Xcode toolkit.
b. In Windows, the code should be compiled using MinGW. The gcc and std c++ libraries should be statically linked. From the MinGW command line, cd into the qSR/SpatialClustering/BioJet/FJCore/fjcore-3.2.0 folder, and then compile by calling

   g++ -static -static-libgcc -static-libstdc++ -O BioJetsTree.cc fjcore.cc -o BioJetsTree

When using either the pair correlation feature[1,5,6] or the FastJet[2-4] hierarchical clustering feature, be sure to cite the original papers in any scientific publication.

Section B: Protocols

B.1: Main Figure 1 Analysis Protocols:

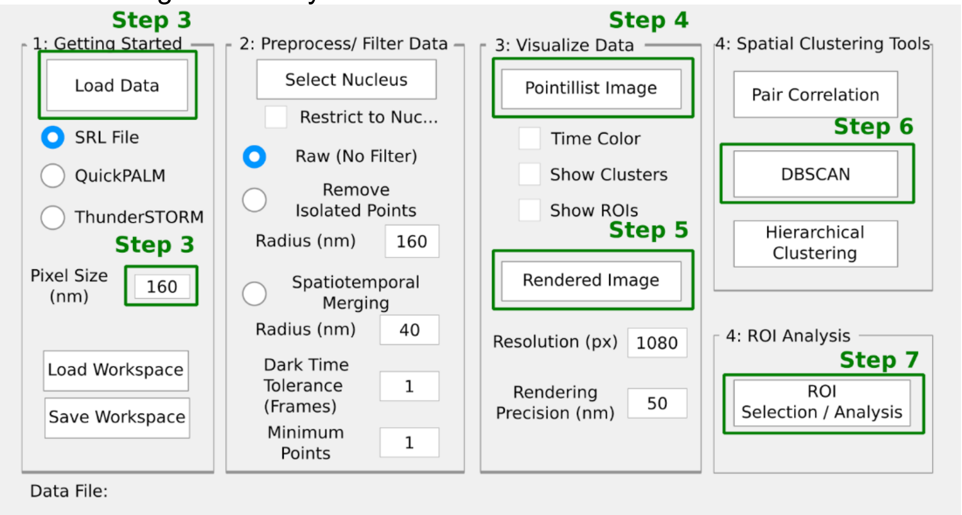

Supplemental Figure 1: The qSR interface.

1. Download and install software from www.github.com/cisselab/qSR
   See installation instructions (in Section A).

2. Run qSR.
   a. Stand alone program: Launch qSR using the desktop shortcut.
   b. From source: Open MATLAB and type qSR into the Command Window and press enter.

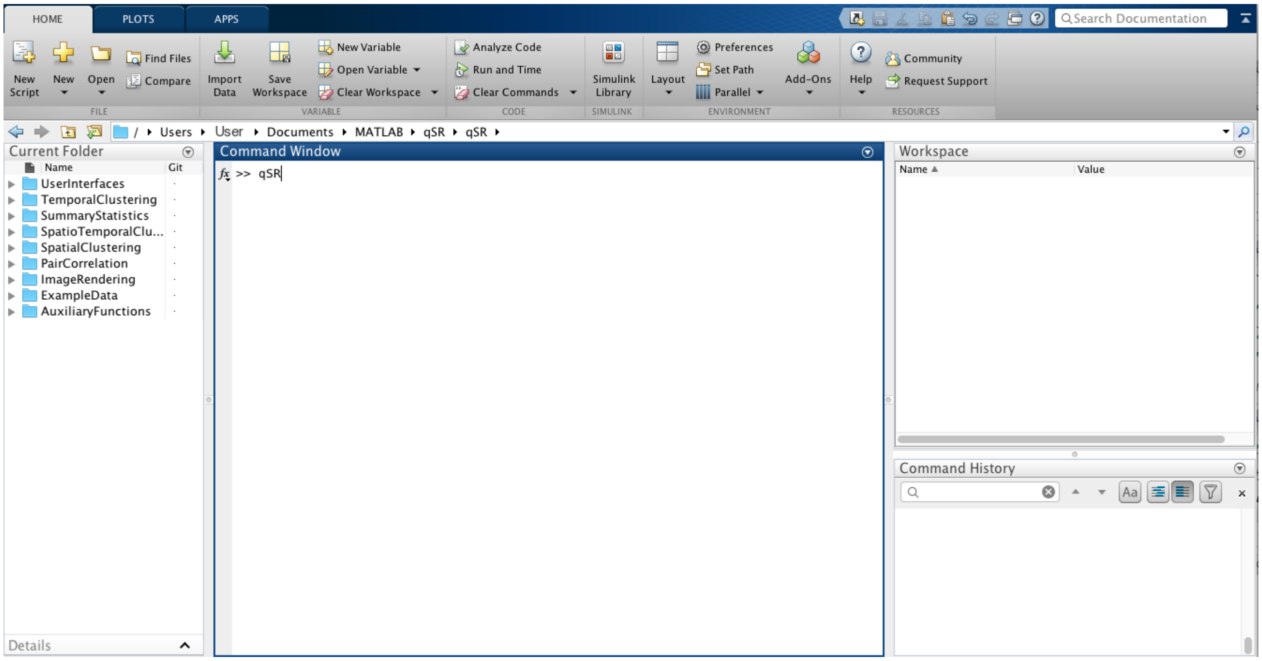

Supplemental Figure 2: Calling qSR from MATLAB.

3. Load a data set. A sample data[7] set is provided in the qSR/ExampleData folder. Set the size of the pixels. For the provided data set, each pixel is 160 nm wide.

4. Clicking on “Pointillist Image” generates Figure 1b.

5. To reproduce the super-resolution image shown in Figure 1c, click “Rendered Image”.

6. Spatial clustering can be performed using one of two algorithms: DBSCAN[8] or FastJet[2-4] hierarchical clustering. To run DBSCAN, click “DBSCAN Clustering”. The parameters used to generate Figure 1e were a Length Scale of 100 nm and a Minimum Points of 8 points. Click “Run” to perform the analysis, and verify the clustering assignment. Clicking “Export Cluster Data” will export the clustering assignment, and the calculated summary statistics; all clustering parameters are automatically saved in the metadata associated with the files. The output csv files may be too large to be opened in Excel. Figure 1f is produces using FastJet; for a detailed protocol, see section B.2.b below.

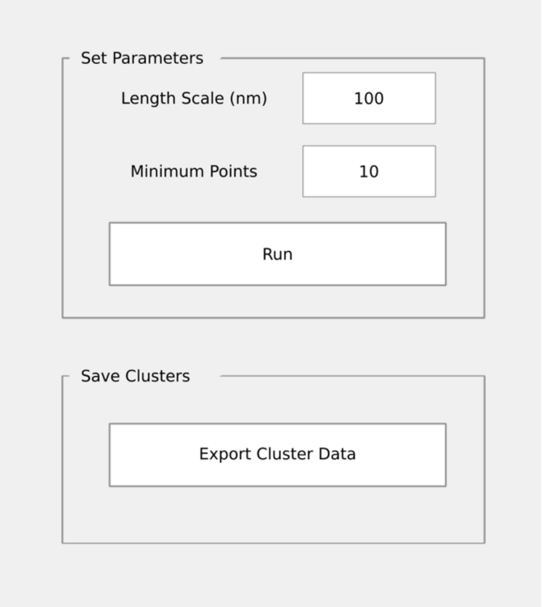

Supplemental Figure 3: DBSCAN interface.

7. To perform temporal clustering analysis[9,10], click “ROI Selection / Analysis”. Clicking “Select New ROI” allows the user to select clusters within the pointillist graph. “Delete Current ROI” will delete the currently selected ROI, and “Select and Delete ROIs” allows the user to draw a rectangle on the pointillist image around the ROIs that they would like to delete. The user can navigate between the ROIs by using the “Previous ROI” and “Next ROI” buttons. Clicking “Subsection Current ROI” allows the user to refine the boundaries of the current ROI by drawing a rectangle within the spatial axes in the top left corner of the Temporal Clustering window. To generate a clustering assignment, set a minimum cluster size, and drag the Dark Time Tolerance slider bar until the highlighted regions contain only those points that are both temporally and spatially clustered. Clicking “Apply to All ROIs” will propagate the specified parameters to all ROIs. Clicking “Export Cluster Data” will export the clustering assignment, and the calculated summary statistics; all clustering parameters are automatically saved in the metadata associated with the files. The output csv files may be too large to be opened in Excel.

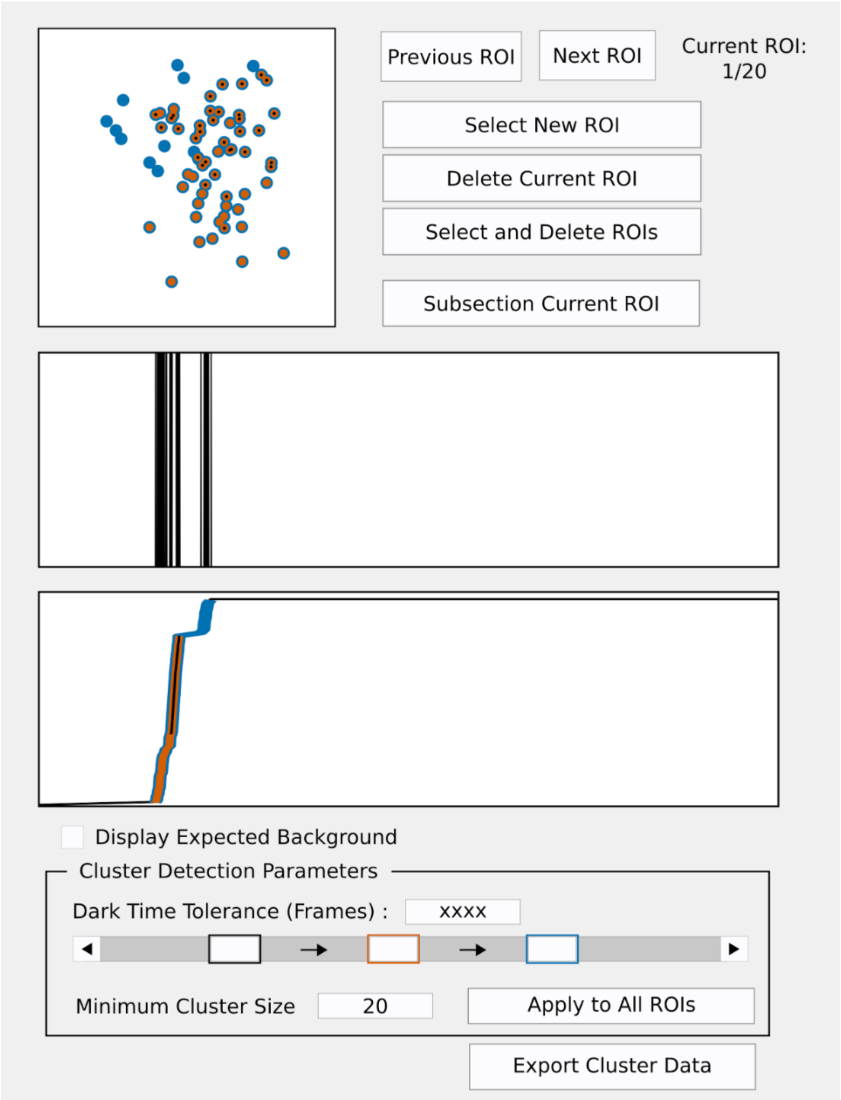

Supplemental Figure 4: Schematic Representation of the temporal cluster selection interface. Cluster assignments are determined by two parameters: Dark Time Tolerance and minimum cluster size. Clusters are generated by grouping the detections into contiguous regions separated by dark times longer than the specified tolerance. The resulting cluster assignments are shown for three example Dark Time Tolerances.

B.2: Additional Analysis Protocols

B.2.a: Pair Correlation Analysis

Pair correlation analysis[1,5,6] is a method to distinguish between single molecule localization patterns, and clustered protein localization patterns. The pair correlation tools in qSR allow the user to filter/preprocess the localization patterns, and generate the pair correlation function of selected regions. For more information on the null model, and how to statistically test for clustering, we refer the user to Sengupta *et al.*[5]

1. Launch qSR.

2. Load Data and set the pixel size.

3. Set the Preprocess/ Filter Data status to “Spatiotemporal Merging” and set the Radius to the average localization precision (assumed 40 nm by default), and the Dark Time Tolerance to 0 Frames. This will combine multiple consecutive localizations from the same single molecule into one localization to reduce overcounting.

4. Click “Pair Correlation”, then click “Select Region”, and select a large, homogenous region (e.g., by avoiding the empty nucleoli in the provided data set).

5. Click “Run Analysis” and then “Export Results”. The results of the analysis are saved within the sample data folder as a csv.

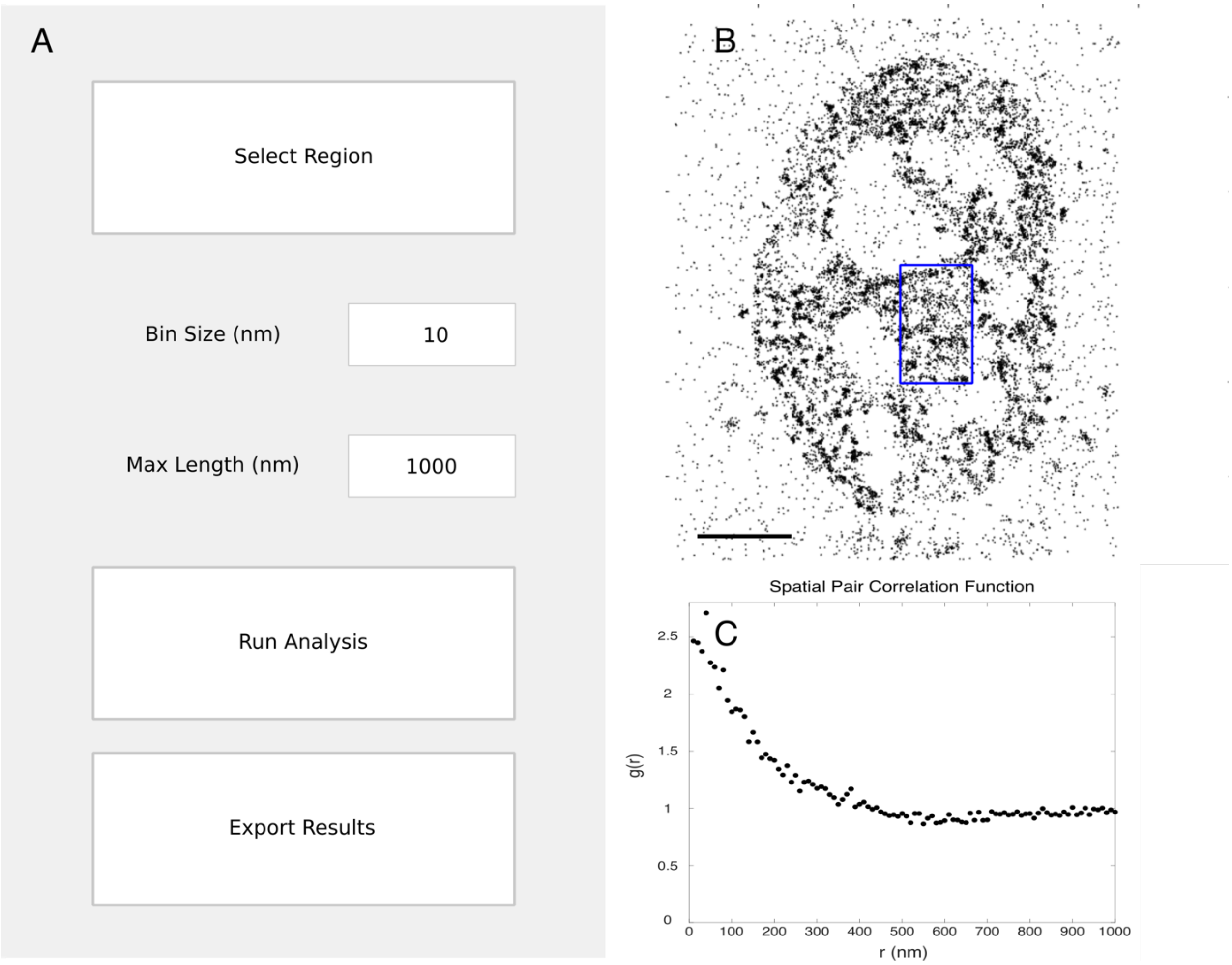

Supplemental Figure 5: Pair correlation analysis. (A) Pair Correlation User Interface. (B) The user selects the region of interest on which to run pair correlation analysis. (C) qSR generates the pair correlation function. Scale Bars – B: 5 μm

B.2.b: FastJet Hierarchical Clustering

FastJet[2] is a software package developed by the particle physics community for the analysis of collision data. This software is used extensively by all four major detectors at the Large Hadron Collider: ALICE, LHCb, ATLAS, and CMS. We make use of the Cambridge-Aachen algorithm[3,4], to generate a pairwise clustering tree. By running the Cambridge-Aachen algorithm with boost-invariant pt recombination scheme, FastJet is effectively computing a hierarchical clustering tree with centroid linkage, with an asymptotic runtime of N log N where N is the number of localizations per cell. After generating the clustering tree, the user can select the cluster length scale and quickly compute the clusters. Creation of the tree requires no input parameters, and the tree can be quickly cut after choosing a length scale and minimum cluster size. By separating these two steps, the user can quickly assess many different clustering parameters without any substantial computational cost.

1. Launch qSR.

2. Load Data and set the pixel size.

3. Click “Hierarchical Clustering”; the clustering tree will be automatically generated.

4. Once the tree is created, different clustering length scales can be tried in quick succession. Set the desired Length Scale and Minimum Size, and click “Find Clusters”. For the user’s convenience, the software shows clusters for the specified length scale, as well as a smaller and a larger length scale, to help the user more quickly determine an appropriate Length Scale.

5. Once an appropriate length scale has been set, click “Export Cluster Data” to export the clustering assignment, and the calculated summary statistics; all clustering parameters are automatically saved in the metadata associated with the files. The output csv files may be too large to be opened in Excel. Click “Save ROIs” to use the FastJet cluster centers to create Regions of Interest for later temporal clustering. In order to uniquely assign points to ROIs, overlapping ROIs are merged. Densely overlapping ROIs may generate excessively large ROIs. To address this, the user is advised to either try a different set of clustering parameters, or to manually select ROIs in the problematic regions, using the “ROI Selection / Analysis” tool in the main qSR interface.

6. If using FastJet to select Regions of Interest, click on “ROI Selection / Analysis” in the main qSR interface and continue with temporal analysis, as in step 7 from section B.1.

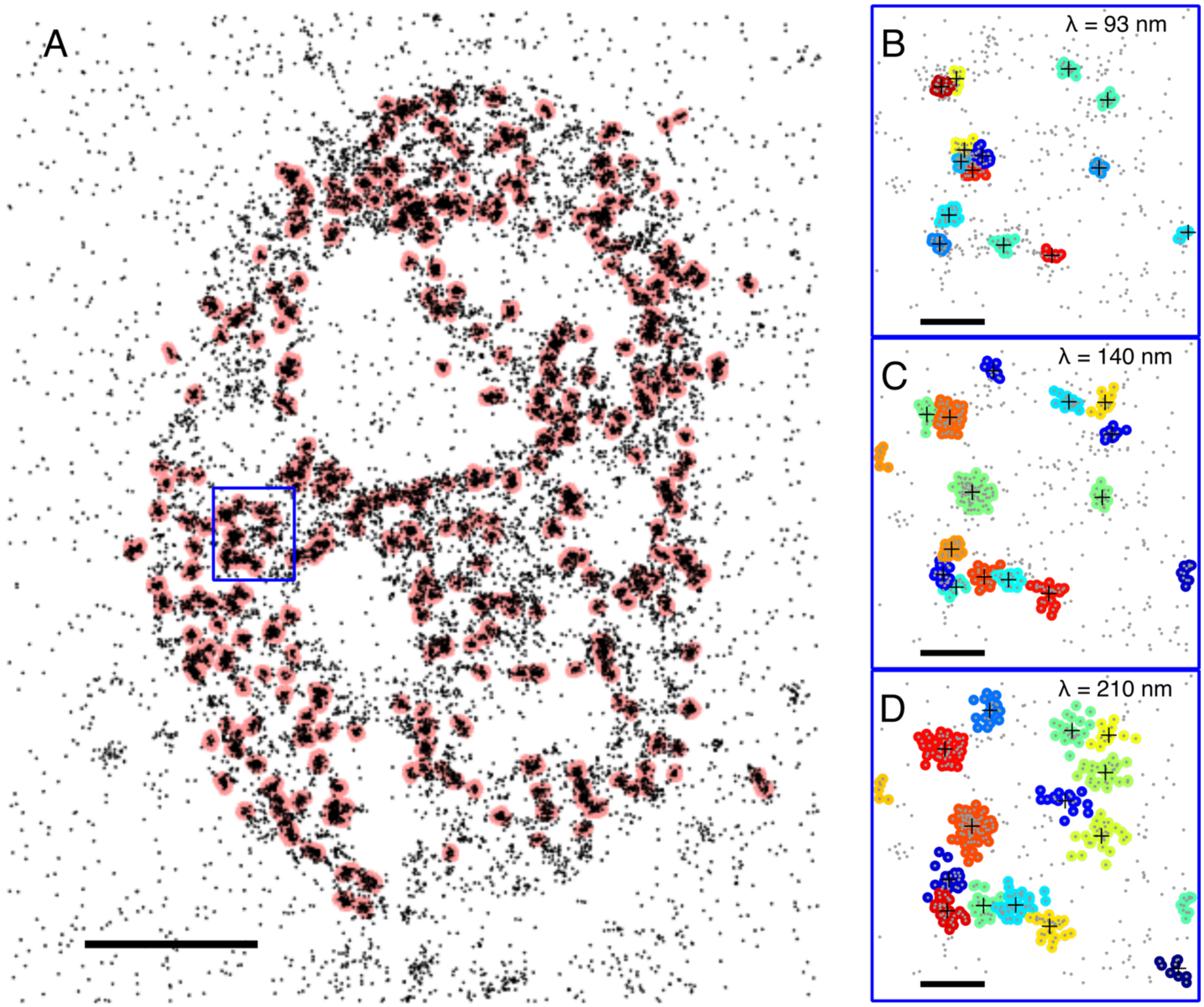

Supplemental Figure 6: FastJet hierarchical clustering. (A) FastJet clusters found with a length scale of 140nm. (B-D) Zoomed in view of the region in the blue box from A. The clusters were generated by cutting the tree with a length scale of 93 nm, 140 nm, and 210 nm respectively. The black + signs mark the centroids of each cluster. Scale Bars – A: 5 μm B - D: 500 nm

B.3: Single Molecule Control Data

In single-molecule based super-resolution microscopy, the dynamic assembly and disassembly of a cluster would appear as a transient increase in the rate of detections localized in a region of interest. Depending on the camera acquisition settings and the fluorophore photophysics, single molecules may give rise to multiple, intermittent localizations. The starting point for any analysis of protein clustering dynamics in super-resolution microscopy should start with characterizing the intrinsic behavior of immobile single molecules. This can be achieved by chemical fixation, or by conjugating the fluorophore to an immobile protein, such as histone protein H2B. The single molecule photophysics depends on the choice of fluorophore (for the provided data sets, we used Dendra2), illumination intensities and the local chemical environment, so control experiments should be performed using the same imaging settings as the primary data.

Along with the software, we provide a control dataset obtained by fixation of cells transiently transfected with free Dendra2. The survival plot (1-CDF, i.e. the complement of the cumulative distribution function) of the burst size is represented in Supplemental Figure 7. The measure mean burst size per Dendra2 molecule is 6.3 localizations; any given molecule has some probability of obtaining an even larger count of detections, with (two) rare events seen to yield as many as 150 localizations. When analyzing clusters, it is beneficial to set a threshold of minimum cluster size to exclude individual molecules. For example, setting a minimum size of 24 counts excludes 95% of single molecules. It is important that reports of transient sub-diffractive clusters be corroborated with biological controls, such as perturbations that change cluster dynamics in a functionally relevant manner (for Pol II, see serum induction or gene co-localization in Cho et al.[10]).

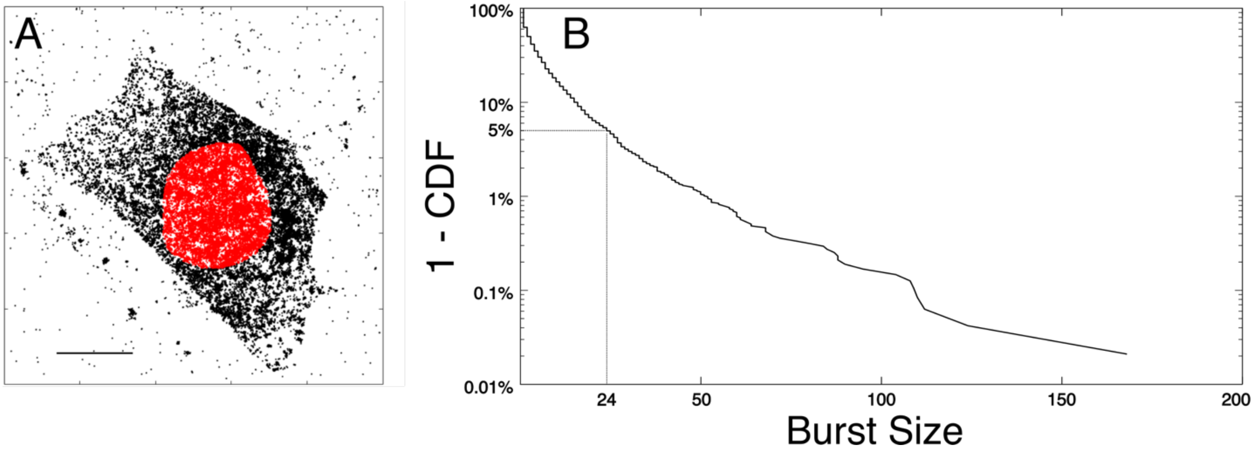

Supplemental Figure 7: Characterizing single molecules. (A) Pointillist representation of the control data. A nuclear section (highlighted in red) is used to analyze the single molecule burst size distribution. (B) The survival plot of the size of bursts in the nuclear region. Scale Bars – A: 8 μm

Section C: Input Data Format

qSR takes as input a list of super-resolution localizations generated by single molecule localization microscopy[11-13]. The software assumes that the data will be in four columns (frames, x position, y position, intensity) with the first row containing a header. To verify that it is in this format, qSR checks for the filename to end in SRL.csv (e.g. cell1_SRL.csv).

Alternatively, data from QuickPALM[14] or ThunderSTORM[15] can also be used by selecting those options before clicking "Load Data" in the qSR user interface. ThunderSTORM data output is assumed to be a csv file with frame number in the first column, x position (in nanometers) in the second column, y position (in nanometers) in the third column, and intensity in the fourth column.

Section D: Software File Structure and Data Representation

qSR was built using MATLAB's GUIDE tool. The user interface can be modified by opening qSR.fig with GUIDE. The source code that determines how qSR functions is located in qSR.m. Callback functions that execute upon pressing buttons in the UI are grouped in qSR.m by the panel in which they are located. Data is stored in the GUI as fields in the handles structure. The raw x position data, for example, can be accessed by calling handles.XposRaw. All functions from panel 3 (Visualize) onward access only the filtered data, e.g. handles.fXpos, which is obtained by scaling to nanometers and applying the appropriate filters.

Buttons that call popup interfaces (pcPALM, DBSCAN, Hierarchical clustering, and Temporal Clustering) create new GUIs with separate handles variables. Data from the main GUI is accessible using mainObject, and mainHandles.

Section E: Summary of Included Algorithms

Algorithm	Inputs	Outputs
Isolated Point Filter	-Neighborhood Size	-All points containing at least one neighboring point in the previous or subsequent frame.
Spatiotemporal Merging	-Radius<br>-Dark Time Tolerance<br>-Minimum Size	-Centroids of clusters of points within the radius with continuous stretches of darkness less that the dark time tolerance.
Pair Correlation	-Region of Interest<br>-Bin Size<br>-Maximum Length	-The pair correlation function between binned data points within the region of interest evaluated up to the maximum length.
DBSCAN	-Length Scale<br>-Minimum Points	-Density-based clusters of points<br>-Number of localizations per cluster<br>-Cluster Areas
FastJet Hierarchical Clustering	-Length Scale<br>-Minimum Points	-Dendrogram of hierarchical clusters.<br>-Clusters of points from centroid-linkage hierarchical clustering at a particular length scale.<br>-Number of localizations per cluster<br>-Cluster Areas
Temporal Clustering	-Regions of Interest<br>-Dark Time Tolerance<br>-Minimum Size	-Spatio-temporal clusters of points<br>-Number of localizations per cluster<br>-Cluster Areas<br>-Cluster Lifetimes

Section F: User Feedback and Bug Reports

Users of the software are invited to ask questions, suggest features, and report bugs by logging issues on www.github.com/cisselab/qSR.

References:

1 Veatch, S. L. *et al.* Correlation Functions Quantify Super-Resolution Images and Estimate Apparent Clustering Due to Over-Counting. *Plos One* **7**, doi:ARTN e31457 10.1371/journal.pone.0031457 (2012).

2 Cacciari, M., Salam, G. P. & Soyez, G. FastJet user manual. *Eur Phys J C* **72**, doi:ARTN 1896 10.1140/epjc/s10052-012-1896-2 (2012).

3 Dokshitzer, Y. L., Leder, G., Moretti, S. & Webber, B. Better jet clustering algorithms. *Journal of High Energy Physics* **1997**, 001 (1997).

4 Wobisch, M. & Wengler, T. Hadronization corrections to jet cross sections in deep-inelastic scattering. *arXiv preprint hep-ph/9907280* (1999).

5 Sengupta, P. *et al.* Probing protein heterogeneity in the plasma membrane using PALM and pair correlation analysis. *Nature methods* **8**, 969-975, doi:10.1038/nmeth.1704 (2011).

6 Sengupta, P., Jovanovic-Talisman, T. & Lippincott-Schwartz, J. Quantifying spatial organization in point-localization superresolution images using pair correlation analysis. *Nature protocols* **8**, 345-354 (2013).

7 Cho, W. K. *et al.* Super-resolution imaging of fluorescently labeled, endogenous RNA Polymerase II in living cells with CRISPR/Cas9-mediated gene editing. *Sci Rep-Uk* **6**, doi:Artn 35949 10.1038/Srep35949 (2016).

8 Ester, M., Kriegel, H.-P., Sander, J. & Xu, X. in *Proceedings of the Knowledge Discovery and Data Mining Conference, AAAI Press.* 226-231.

9 Cisse, II *et al.* Real-time dynamics of RNA polymerase II clustering in live human cells. *Science (New York, N.Y.)* **341**, 664-667, doi:10.1126/science.1239053 (2013).

10 Cho, W. K. *et al.* RNA Polymerase II cluster dynamics predict mRNA output in living cells. *Elife* **5**, e13617, doi:ARTN e13617 10.7554/eLife.13617 (2016).

11 Betzig, E. *et al.* Imaging intracellular fluorescent proteins at nanometer resolution. *Science* **313**, 1642-1645, doi:10.1126/science.1127344 (2006).

12 Hess, S. T., Girirajan, T. P. & Mason, M. D. Ultra-high resolution imaging by fluorescence photoactivation localization microscopy. *Biophysical journal* **91**, 4258-4272, doi:10.1529/biophysj.106.091116 (2006).

13 Rust, M. J., Bates, M. & Zhuang, X. Sub-diffraction-limit imaging by stochastic optical reconstruction microscopy (STORM). *Nature methods* **3**, 793-795, doi:10.1038/nmeth929 (2006).

14 Henriques, R. *et al.* QuickPALM: 3D real-time photoactivation nanoscopy image processing in ImageJ. *Nature methods* **7**, 339-340 (2010).

15 Ovesný, M., Křížek, P., Borkovec, J., Švindrych, Z. & Hagen, G. M. ThunderSTORM: a comprehensive ImageJ plug-in for PALM and STORM data analysis and super-resolution imaging. *Bioinformatics* **30**, 2389-2390 (2014).
